# Neuropsychological profile, quantitative MRI data and brain‐derived circulating cell‐free DNA in plasma: a cross‐sectional study of the Alzheimer’s disease continuum

**DOI:** 10.1002/alz.095222

**Published:** 2025-01-09

**Authors:** Micaela Mitolo, Maria Giulia Bacalini, Elena Cantoni, Giovanni Sighinolfi, Luisa Sambati, Lucia Guidi, Virginia Pollarini, Rossella Santoro, Luca Morandi, Camilla Pellegrini, Francesco Ravaioli, Chiara Pirazzini, Riccardo Manca, Annalena Venneri, Maria Guarino, Pietro Cortelli, Raffaele Lodi, Caterina Tonon

**Affiliations:** ^1^ IRCCS Istituto delle Scienze Neurologiche di Bologna, Bologna, Italy Italy; ^2^ University of Parma, Parma Italy; ^3^ IRCCS Istituto delle Scienze Neurologiche di Bologna, Bologna Italy; ^4^ IRCCS Istituto delle scienze neurologiche di Bologna, Bologna, Italy Italy; ^5^ IRCCS Istituto delle Scienze Neurologiche di Bologna, Bologna, Bologna Italy; ^6^ Policlinico Sant'Orsola Malpighi, Bologna, Italy Italy; ^7^ Brunel University London, Uxbridge United Kingdom; ^8^ Brunel University London, London United Kingdom; ^9^ University of Bologna, Bologna, italy Italy; ^10^ University of Bologna, Bologna Italy

## Abstract

**Background:**

Cognitive decline in Alzheimer’s disease (AD) continuum is knowingly associated with degenerative neurobiological features; however, reliable markers for the early detection of the preclinical stages of AD are not fully established. The main goal of this study was to track ongoing brain neurodegeneration across the AD continuum by combining neuropsychological data, brain MRI features, and plasmatic ‐ brain‐derived circulating cell‐free DNA (b‐cfDNA) biomarkers.

**Method:**

A total of 91 participants were included in this study including 28 healthy controls (HC) (mean age 66.4±9.2), 21 participants with Subjective Cognitive Decline (SCD) (mean age 70.4±8.5), 18 participants with Mild Cognitive Impairment (MCI) (mean age 72.3±7.1) and 24 patients with AD dementia (mean age 71.5±8.3). All participants underwent a neuropsychological evaluation, a brain MRI and a blood sample test to quantify the levels of brain‐derived cfDNA in plasma. ANCOVAs were used to test differences across participants. In addition, the median value of b‐cfDNA levels was set as a threshold to categorize participants into b‐cfDNA‐positive or negative. Pearson’s correlations were carried out between all variables. Results were corrected for multiple comparisons and statistical significance was set at p<0.05.

**Result:**

Several regions in frontal, temporal and inferior parietal lobes showed progressively lower volumes going from HC to AD dementia. Although no statistical differences between mean b‐cfDNA levels were observed among groups, we found a trend of increased values in SCD and MCI participants. In addition, volumes of specific temporal regions differed significantly between b‐cfDNA‐positive and b‐cfDNA‐negative participants. Moreover, b‐cfDNA values correlated with semantic fluency scores in the AD dementia group (p = 0.038) and with the Stroop test scores in the HC group (p = 0.007).

**Conclusion:**

These results suggest that active neurodegeneration in the early phases of AD can be associated with a release of b‐cfDNA in plasma. Thus, this blood‐based biomarker might reveal early neurodegenerative processes even in the pre‐clinical dementia stage, when the neuropsychological and MRI changes are very subtle and difficult to detect at the individual level.

**Acknowledgement**: This study is supported by funding obtained under the National Recovery and Resilience Plan (NRRP), Mission 4 Component 2 Investment 1.3 ‐Project code PE0000006, CUP D93C22000930002, MNESYS

